# Supplementary material for: (E)-2-Cyano-3-(1H-Indol-3-yl)-N-Phenylacrylamide, a Hybrid Compound Derived from Indomethacin and Paracetamol: Design, Synthesis and Evaluation of the Anti-Inflammatory Potential
Source: Int J Mol Sci. 2020 Apr 8;21(7):2591. doi: 10.3390/ijms21072591 (PMC7177726; doi:10.3390/ijms21072591)

# (E)-2-cyano-3-(indol-3-yl)-N-phenylacrylamide, a Hybrid Compound Derived from Indomethacin and Paracetamol: Design, Synthesis and Evaluation of Anti-inflammatory Potential

**Pablo Silva, Maria de Almeida, Jamire Silva, Sonaly Albino, Renan Espirito-Santo, Maria Lima, Cristiane Villarreal, Ricardo Moura, Vanda Santos.**

## Supplementary data

*Supplementary data captions*

**Figure S1** FT-IR spectrum of **ICMD-01**

**Figure S2** $^1$H NMR spectrum of **ICMD-01**

**Figure S3** $^{13}$C NMR spectrum of **ICMD-01**

**Figure S4** HRMS m/z [$M^+$ + Na] of **ICMD-01**

**Figure S5** 3D crystallographic pose of co-cristallized ligand indomethacin (yellow) and redocked ligand (gray) on binding site of cyclooxygenase-2 (4COX)

**Figure S1.** FT-IR spectrum of **ICMD-01**

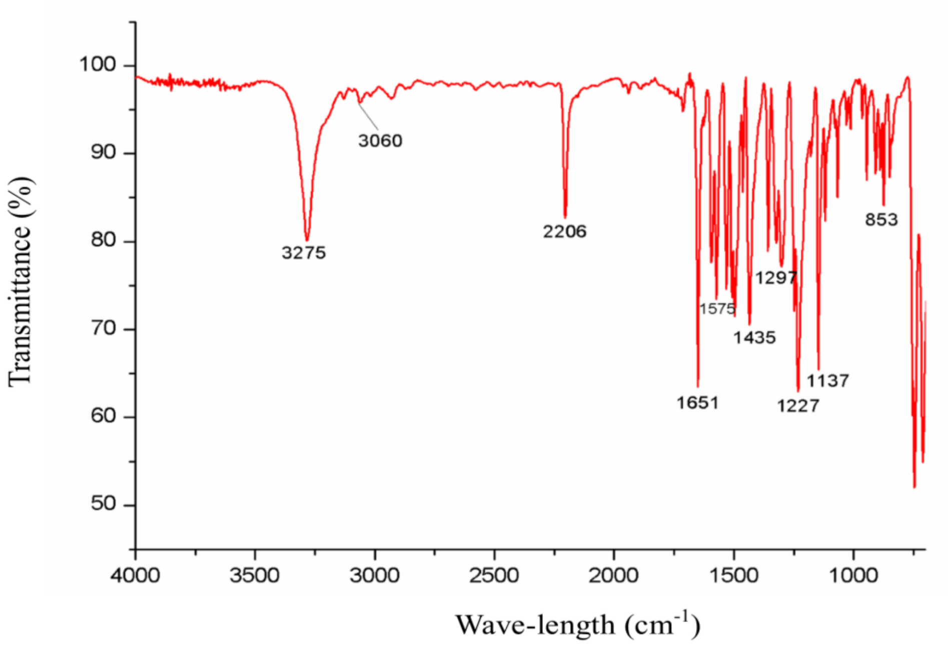

**Figure S2.** $^1$H NMR spectrum of **ICMD-01**

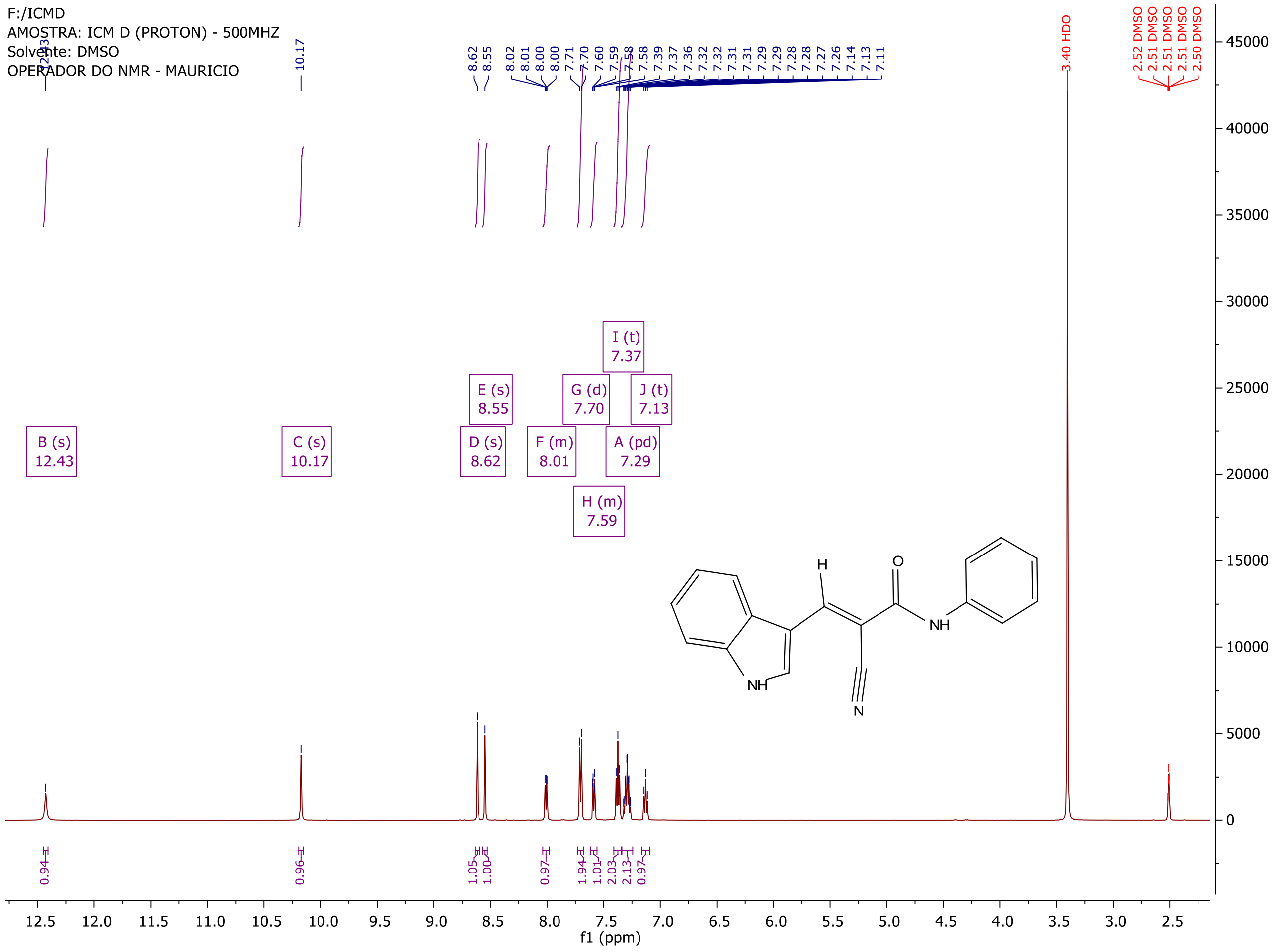

**Figure S3.** $^{13}$C NMR spectrum of **ICMD-01**

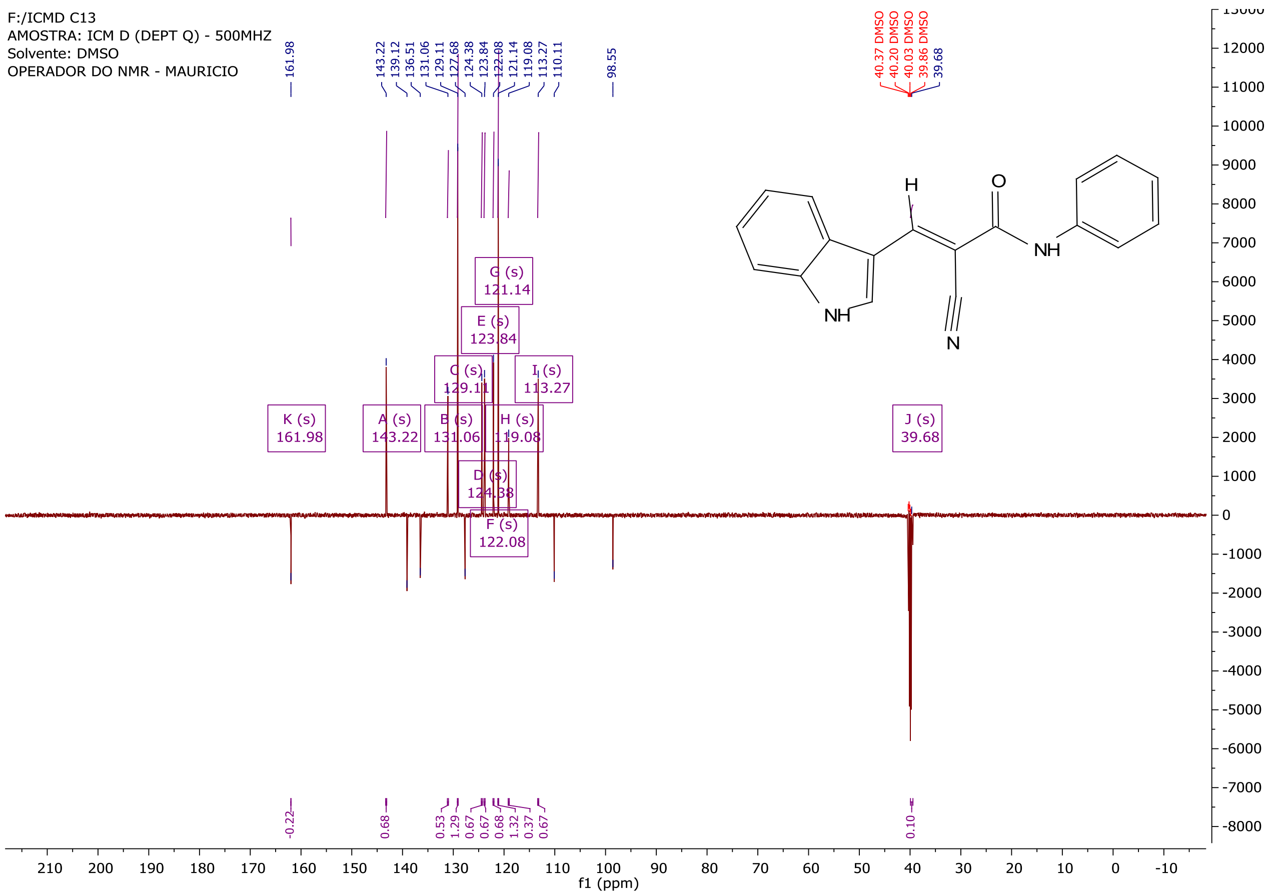

**Figure S4**. HRMS m/z [$M^+$ + Na] of **ICMD-01**

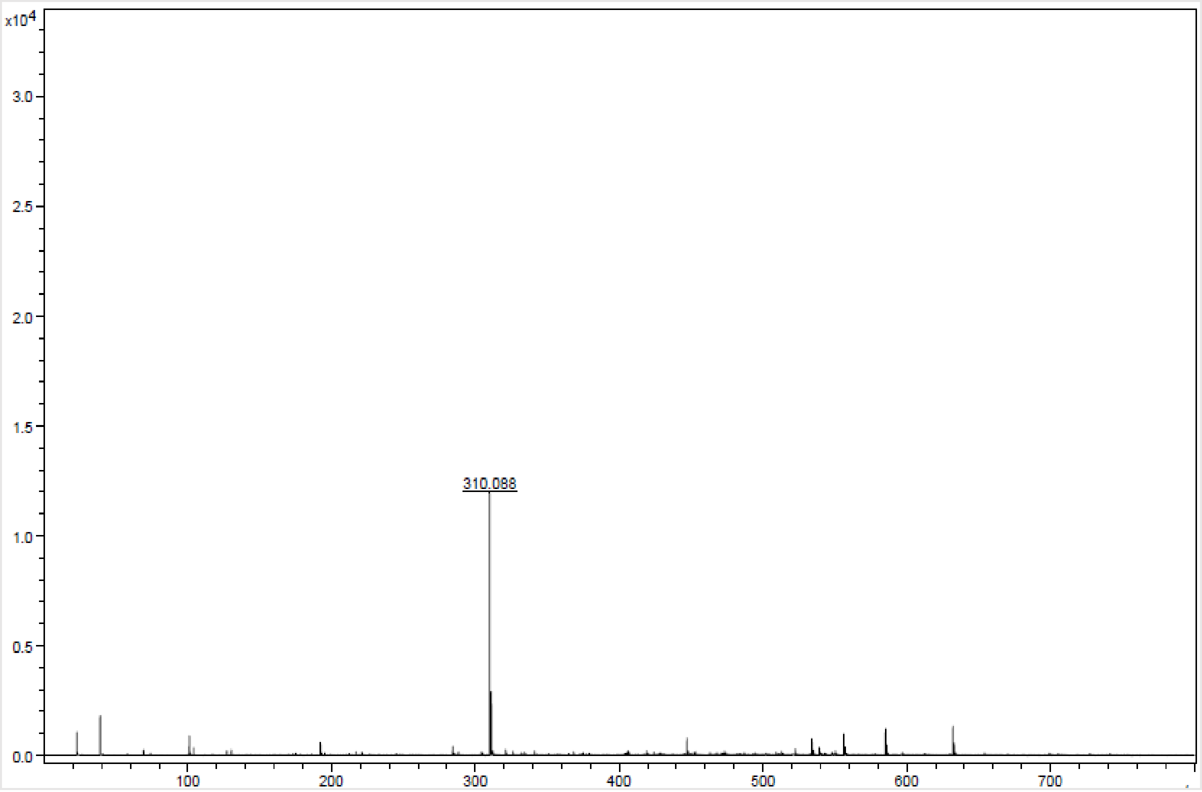

**Figure S5.** 3D crystallographic pose of co-cristallized ligand indomethacin (yellow) and redocked ligand (gray) on binding site of cyclooxygenase-2 (4COX)

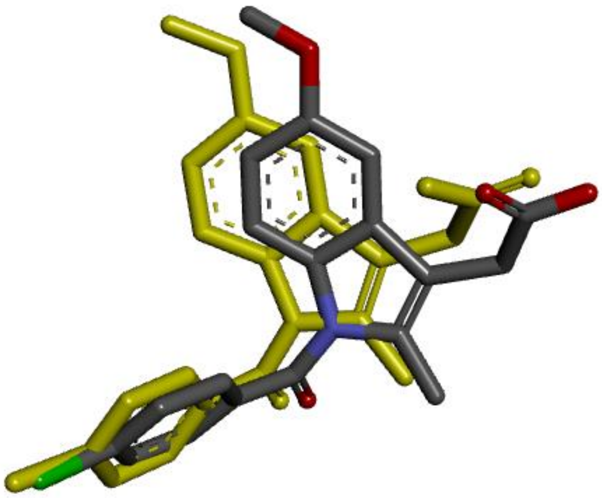

Supplement: Supplementary file 1 [file ijms-21-02591-s001.pdf]
